# Protocol for a cross-sectional study on COVID-19 vaccination programmes in primary health care

**DOI:** 10.4102/phcfm.v15i1.3649

**Published:** 2023-01-31

**Authors:** Sumeet Sodhi, Rifka Chamali, Devarsetty Praveen, Manushi Sharma, Marcelo Garcia Dieguez, Robert Mash, Felicity Goodyear-Smith, David Ponka

**Affiliations:** 1 Besrour Centre for Global Family Medicine, College of Family Physicians of Canada, Mississauga, Canada; 2 Toronto Western Hospital, University Health Network, Toronto, Canada; 3 Department of Family and Community Medicine, Faculty of Medicine, University of Toronto, Toronto, Canada; 4 The George Institute for Global Health India, New Delhi, India; 5 University of New South Wales, Sydney, Australia; 6 Prasanna School of Public Health, Manipal Academy of Higher Education, Manipal, India; 7 Centre for Study of Health Professions Education, National University of the South, Bahia Blanca, Argentina; 8 Division of Family Medicine and Primary Care, Faculty of Medicine and Health Sciences, Stellenbosch University, Stellenbosch, South Africa; 9 Department of General Practice and Primary Health Care, Faculty of Medical and Health Sciences, University of Auckland, Auckland, New Zealand; 10 Department of Family Medicine, Faculty of Medicine, University of Ottawa, Ottawa, Canada

**Keywords:** integrated health systems, primary health care, primary care, public health, immunisation, global health, COVID-19

## Abstract

**Background:**

An integrated primary health care approach, where primary care and public health efforts are coordinated, is a key feature of routine immunisation campaigns.

**Aim:**

The aim of the study is to describe the approach used by a diverse group of international primary health care professionals in delivering their coronavirus disease 2019 (COVID-19) vaccination programmes, as well as their perspectives on public health and primary care integration while implementing national COVID-19 vaccination programmes in their own jurisdictions.

**Setting:**

This is a protocol for a study, which consists of a cross-sectional online survey disseminated among a convenience sample of international primary health care professional through member-based organisations and professional networks via email and online newsletters.

**Methods:**

Survey development followed an iterative validation process with a formative committee developing the survey instrument based on study objectives, existing literature and best practices and a summative committee verifying and validating content.

**Results:**

Main outcome measures are vaccination implementation approach (planning, coordination service deliver), level or type of primary care involvement and degree of primary care and public health integration at community level.

**Conclusion:**

Integrated health systems can lead to a greater impact in the rollout of the COVID-19 vaccine and can ensure that we are better prepared for crises that threaten human health, not only limited to infectious pandemics but also the rising tide of chronic disease, natural and conflict-driven disasters and climate change.

**Contribution:**

This study will provide insight and key learnings for improving vaccination efforts for COVID-19 and possible future pandemics.

## Introduction

Primary health care is a:

Whole-of-society approach to health that aims to ensure the highest possible level of health and wellbeing and their equitable distribution by focusing on people’s needs and preferences as early as possible along the continuum from health promotion and disease prevention to treatment, rehabilitation and palliative care, and as close as feasible to people’s everyday environment.^1^ (p. 2)

An essential component of effective Primary health care (PHC) involves the integration of primary care for individuals and families and public health services for populations. Primary care and public health services have overlapping functions and can achieve greater outcomes by their integration and coordination.^2,3^

Studies show that primary care and public health integration in immunisation programmes lead to successful delivery and wider national coverage.^3,4,5,6^ Primary care providers (PCPs) play a key role in successfully delivering childhood and adult vaccinations.^6,7^ In addition, they are a trusted source for vaccine information, build local community trust, reach vulnerable and marginalised communities and contribute to good uptake.^6,8,9^ Public health has a key role in establishing the effectiveness and safety of vaccines, responding to outbreaks, setting vaccine schedules and vaccine policies.^10^ Primary care and public health integration in immunisation programmes for influenza^4,6,9,11^ (including swine flu [H1N1]^8^), human papilloma virus (HPV)^12^ and meningococcal B disease proved to be successful.^13^ For example, PCPs were instrumental in improving national coverage for the influenza vaccine in the United Kingdom and the United States, the H1N1 vaccine in Europe and the HPV in low- and middle-income countries, by championing active outreach to at-risk and marginalised communities.^4,8,9,11,12^

Another classic integration approach that facilitates primary care and public health services is co-location, which can increase efficiency and uptake of vaccine programmes; for example, by integrating administration of routine vaccinations with regular primary care clinical visits.^12^ In Cameroon, more efficient health service delivery was achieved by offering HPV vaccines during cervical cancer screening appointments in a primary care facility.^13^ A similar improvement in vaccine uptake was noted in Italy by offering concurrent counselling and vaccine administration for meningococcal B disease during a regular primary care visit.^14^

Immunisation against coronavirus disease 2019 (COVID-19) is a key factor in controlling the COVID-19 pandemic.^15^ The challenge remains to ensure equitable distribution of vaccines between and within countries.^16^ In addition to helping to achieve national vaccination coverage, coordinated and integrated immunisation services have been shown to strengthen PHC, facilitate progress towards universal health coverage and contribute to achieving health-related sustainable development goals. Integrated PHC allows a health system to address different communities and vulnerable groups within a society and achieve vaccine accessibility for all.^17^ To date, while there is literature that highlights primary care’s potential role in COVID-19 vaccination campaigns,^6,9^ there is a gap in understanding the actual implementation approaches used in different PHC contexts.

The overall aim of the study is to describe the approach utilised by a diverse group of international PHC professionals in different countries in delivering their COVID-19 vaccination programmes, as well as their perspectives on public health and primary care integration while implementing national COVID-19 vaccination programmes in their own jurisdictions. In order to understand the different PHC contexts, a global approach across different countries with low-, middle- and high-income countries (HICs) included. The specific research objectives are to:

- Describe the implementation approach for the COVID-19 vaccination programmes, with a focus on planning, coordination and service delivery across different countries and contexts.
- Understand the level and type of primary care involvement in the delivery of COVID-19 vaccination programmes across different countries and contexts.
- Assess perceptions of primary care and public health integration in terms of primary care attributes, population health management approaches and implementation outcomes for the COVID-19 vaccination programmes, from the perspective of PHC professionals.

## Research methods and design

### Study design and setting

This is a protocol for a study, which consisted of a cross-sectional online survey to be disseminated to a convenience sample of international PHC professionals through member-based organisations and professional networks over 4 months (November 2021 to February 2022) by email and online newsletters. Reminder were sent twice within a three week interval. In order to accommodate regional linguistic differences around the world, the survey and study participant materials (invitation, informed consent form) were translated from English into the other official languages of the United Nations (Arabic, Chinese, French, Russian, Spanish) as well as Portuguese to allow for greater representation and decrease selection bias related to language.

### Study population and sampling strategy

Participants were eligible to participate if they were 18 years or over, self-define themselves as a PHC professional and are able to complete the survey in one of the available languages. Primary health care professionals include academics, researchers, government staff, policymakers and PCPs such as general practitioners, family doctors, mid-level health care providers, nurses and community pharmacists. Other specialties or tertiary care professionals are not eligible to participate, although if a professional in public health self-identified as a PHC professional, they were not excluded.

Recruitment was based on a voluntary, convenience sample of PHC professionals. The researchers targeted established PHC professional networks and member-based organisations in different regions of the world, which are within the partner and collaborator associated with the Besrour Centre for Global Family Medicine at the College of Family Physicians of Canada and the Primary Health Care Research Consortium at the George Institute for Global Health (see Appendix A for a full list). Networks and organisations disseminated a letter of invitation by email, text or through their social media accounts (see Appendix B for letter). The letter of invitation contained brief details on the research study and a website link that directed potential participants to the informed consent page where participants had the opportunity to read the study details in greater depth and decide whether they want to participate. If they agreed, they were prompted to start the survey. Participants had the opportunity to enter a prize draw for an e-book entitled *How to Do Primary Care Educational Research*. Once the survey was closed, 20 participants were selected randomly to receive the book. Our aim is to recruit a minimum of 300 participants, with a goal to recruit at least 5 participants from 30 different countries. A minimum of five participants for inclusion at the country-level analysis was deemed by the authors to be sufficient saturation based on our previous work.^18^

### Survey development

During the development of the survey, the research team, comprising global PHC professionals, was split into a formative and summative committee. The formative committee (S.S., D.Ponka., R.C.) created the survey questions based on study objectives and existing literature.^19,20,21,22,23^ The study development resources for individual questions were based on World Health Organization (WHO) frameworks for monitoring and evaluating vaccination programmes and evidence-based health systems indicators for integrating primary care and public health. The summative committee (F.G-S., R.M., D.Praveen., M.D.G., M.S.) verified and approved the questions for validity using their expert knowledge and feasibility for online data collection. The survey underwent an iterative process until the final version was approved by both committees. In this process, the research team wanted to ensure that the survey was brief (less than 15 min, 27 questions) using adaptive questions when possible (questions conditionally displayed based on previous responses), three to eight items per page, maximum of five screens, specific to study objectives and applicable to an international and contextually diverse audience.

### Data collection

The survey was hosted on Qualtrics XM and open to anyone who has a weblink to participate. Each survey response was completely anonymous, and no identifiable personal information was collected. Survey data were stored in a password-protected file within a server secure environment. Participants had the option of providing their contact information should they wish to be entered in the prize draw or to receive a summary of the results, but these responses were not linked to the survey responses in any way. Contact information was initially stored in a password-protected file within a server secure environment and was permanently deleted after prize winners were identified and after results were disseminated. Access to contact information was restricted to team members who were contacting prize winners and disseminating the results. Participants can withdraw from the study at any time by exiting the survey and closing their browser. As no identifiable data were collected, participants were unable to withdraw consent after completing the survey.

Participants were able to review and change their responses through the ‘back’ button. Researchers opted not to have forced responses in case participants preferred not to answer a specific question. Participants were, however, reminded of any missed questions and had the option to either answer or proceed with the survey. In order to ensure unique visitors are completing the survey, a cookie is placed in the participant’s browser when responses are submitted, preventing them from taking the survey again. Location and Internet Protocol (IP) addresses were not collected.

### Data analysis

Our main objective is to provide a descriptive analysis of outcomes and conduct comparative analyses by country and region. For country-level analyses, any country that has five or more individual participants responding was included in the analysis. Data were analysed in the Qualtrics XM analytic tool and Microsoft Excel for data cleaning and with SPSS version 28 for data analysis. Descriptive and correlation analysis was conducted for quantitative data and thematic analysis for qualitative data.

We triangulated data related to national vaccination rates, COVID-19 cases and COVID-19 mortality for each country using open-access, publicly available websites.^24,25,26,27^ Demographic variables to be included in the analysis are gender, profession, years in practice and country of residence. Implementation approach to COVID-19 vaccine programmes was inferred through planning, coordination and service delivery outcomes, including type of COVID-19 vaccine use, vaccine registration process and tracking, location of vaccine administration, cost of vaccination from the recipient’s perspective, access among priority groups, barriers to implementation and perceptions of effectiveness of vaccine programme strategies, including equity of access among priority groups and barriers to implementation. The role of primary care in the COVID-19 vaccination campaigns was addressed through indicators for the level and type of primary care involvement such as role, extent and location of vaccination administration by a PCP and provider perspectives on best practices and suggestions for improvement on vaccine programme rollout. Primary care and public health integration was measured by examining attributes of the PHC systems, such as health system infrastructure (use of a unique patient identifier across health care setting and access to immunisation registries), strategy in place to assign patients to a primary care facility, type and scope of care provided to the community, gatekeeper role of primary care, as well as perceptions on the degree of community centeredness and involvement.

### Limitations

Because of the sampling method of a convenience sample and enquiring about past events in the survey, and our use of online data collection, our main study limitations were self-selection (sampling, response and nonresponse) bias, recall bias and order bias. Also, as we have a lack of denominator, an *a priori* sample size and a response rate were not be able to be calculated. As we were not able to determine *a priori* sample size from the standard methods, we elected to determine sample size by *a priori* assessment of saturation – only countries that have five or more responses were included in analyses.

### Ethical considerations

Ethics approval was granted from the Health Sciences Research Ethics Board of the University of Toronto (Protocol number 27873).

## Discussion

An integrated PHC approach, where primary care and public health efforts are coordinated, is a key feature of routine immunisation campaigns and can lead to greater impact in the rollout of the COVID-19 vaccine. Primary care providers can help reduce vaccine hesitancy through the existing trusted and continuous relationships between PCPs and the communities they serve.^5,28,29^ During the planning phase of a vaccine rollout, coordinating with primary care can provide key information on the communities they serve.^28^ In the implementation phase, PCPs, both those based in a facility or those involved in community outreach, can be used as primary vaccinators.^28^ Additionally, an integrated information system is needed at both the population and individual level to verify coverage and monitor vaccine implementation. This will enable providers to provide continuity of care, such as to track second doses or side effects.^28^

An equitable and globally implemented vaccination programme is the only long-term solution to ending the coronavirus disease 2019 pandemic.^15^ As of February 2022, there are 33 vaccines in use and approximately 53% of the world population is fully vaccinated.^24,30^ The situation is starkly different depending on context. In low-income countries (LICs), vaccine access is low, and only 6% of people are fully vaccinated, whereas 72% of the population is fully vaccinated in HICs.^24,26,31^

Contextual differences both add challenge and importance to this study. Creating a survey for a global audience of PCPs requires representation from all continents and across many differing models of care. Differences in terminology,^32^ approaches to COVID-19 and overarching models of care between countries led to a detailed and iterative process of survey creation. Comparing the PHC approach across different contexts allowed us to identify similarities as well as gaps among health systems. This may help identify which factors affect the outcome of vaccination campaigns and can inform future strategies for improvement. In some contexts, integration between PC and PH is assumed, and questions probing this were seen as redundant. It was interesting to see whether such models have led to more successful vaccination campaigns.

Further lessons from a methodological point of view have to do with granularity at the country level. While maintaining our level of analysis at this level, we were compelled to probe around sub-national practices where this was especially relevant either for political or structural reasons. Thus, respondents in China were able to indicate if they were from Hong Kong, for instance, because of differing approaches and containment seen during the pandemic but also to account for regional representation in populous regions.^33^ However, we were not able to account for regional representation globally. Reconciling and understanding sub-national approaches during the pandemic remain an area of urgent interest, especially for countries where health decisions are devolved to the regional level.^34^

## Conclusion

Humanity faces a double imperative during the COVID-19 pandemic. We are collectively looking for opportunities for health system renewal all while living with the increased surge in workload that both the acute and chronic waves of the pandemic bring. As we vacillate between infectious waves and impacts on chronic disease, mental health and social organisation, we need to ensure health systems have the integration and flexibility to respond and to shift between acute and chronic needs for years to come.

Our work aims to examine one aspect of health system integration: the role of primary care and public health in coordinating a rapid, efficient and equitable COVID-19 vaccine distribution. By comparing responses across contexts, we hope to draw lessons that can be adopted widely. These lessons around the integration of primary care and public health can in turn inform approaches to other challenges, beyond the immediate COVID-19 crisis.

As the world moves towards widespread vaccination and immunity, integrated health systems will ensure that we are better prepared for future crises that threaten human health, not only limited to infectious pandemics but also the rising tide of chronic disease, natural and conflict-driven disasters and climate change.

## Appendix A

### List of primary health care professional networks and member-based organizations targeted to help distribute the survey.

1. Afro primary health care (PHC)
2. Ariadne Labs
3. Asociacion Metropolitana de Medicina Familiar
4. Australasian Association for Academic Primary Care
5. College of Family Physicians of Canada
6. Dirección de Formación y Educación Permanente Ministerio de Salud de la Prov de Buenos Aires
7. European Forum for Primary Care
8. European General Practice Research Network
9. Family Physician Forum for Cape Town
10. Federacion Argentina de Medicina Familiar y General
11. George Institute for Global Health
12. Institute of Disaster Preparedness and Response in Hong Kong
13. International Council of Nurses
14. North American Primary Care Research Group
15. Primafamed
16. Primary Health Care Research Consortium
17. Robert Graham Center
18. South African Pharmacy Council
19. The George Institute, Australia
20. The George Institute, India
21. The South African Academy of Family Physicians
22. Universidad Nacional del Sur
23. World Organization of Family Doctors (WONCA)
24. WONCA – Working Party on Research
25. WONCA – Young Doctors

## Appendix B

### Letter of invitation.

Dear [name of individual or organization],

We are pleased to invite you to partake in the ***FM Vax: An International Survey on the Integration of Public Health and Primary Care in COVID-19 Vaccination Campaigns*** research study. The aim is to identify the primary health care approach used by different countries in implementing their vaccination programmes for COVID-19, with a focus on the integration of public health and primary care.

We are interested in input from primary health care professionals, such as clinicians, researchers and policymakers who can respond regarding their own countries. We kindly request your participation by completing a survey that should not take more than 15 min to complete.

You will have the option to enter a prize draw to win an e-book entitled How *t*o Do Primary Care Educational Research.

We welcome you to learn more about the research study and participating by clicking here!

On behalf of the research team, Drs. Sumeet Sodhi, David Ponka, Bob Mash, Praveen Devarsetty, Marcelo Garcia Dieguez and Felicity Goodyear-Smith, our sincere appreciation.

Sincerely,

FM Vax Research Team
